# Alpha-fetoprotein level as a biomarker of liver fibrosis status: a cross-sectional study of 619 consecutive patients with chronic hepatitis B

**DOI:** 10.1186/1471-230X-14-145

**Published:** 2014-08-16

**Authors:** Yu-rui Liu, Bin-bin Lin, Da-wu Zeng, Yue-yong Zhu, Jing Chen, Qi Zheng, Jing Dong, Jia-ji Jiang

**Affiliations:** 1 Center for Liver Disease, First Affiliated Hospital of Fujian Medical University, Fuzhou 350005, Fujian, China; 2 The Second Division of liver diseases, Xiamen Traditional Chinese Medicine Hospital, Xianyue Road, No. 1739, Huli District, Xiamen City, Fujian Province 361000, China

**Keywords:** Alpha-fetoprotein, Fibrosis, Hepatitis B, Chronic, Inflammation

## Abstract

**Background:**

Hepatitis B virus (HBV) infection is a serious public health problem worldwide. This study aimed to investigate the relationship between serum alpha-fetoprotein (AFP) levels and pathological stages of liver biopsy in patients with chronic hepatitis B (CHB).

**Methods:**

The study included 619 patients who were diagnosed with CHB from March 2005 to December 2011. AFP levels were measured by electrochemiluminescence. Liver biopsy samples were classified into five levels of inflammation (G) and fibrosis (S) stages, according to the Chinese guidelines for prevention and treatment of viral hepatitis. Two multivariable ordinal regression models were performed to determine associations between AFP, GGT, and APRI (AST/PLT ratio) and stages of inflammation and fibrosis.

**Results:**

Significant positive and moderate correlations were shown between AFP levels and inflammation stages and between AFP levels and fibrosis stages (ρ = 0.436 and 0.404, p < 0.001). Median values of AFP at liver fibrosis stages S0-1, S2, S3, and S4 were 3.0, 3.4, 5.4, and 11.3 ng/ml, respectively, and median APRI (AST/PLT ratio) was 0.41. Receiver operating characteristic (ROC) curve analyses revealed that the areas under the curves (AUCs) were 0.685, 0.727, and 0.755 (all p <0.001) for judging inflammation stages of G ≥ 2, G ≥ 3, G = 4 by AFP; and 0.691, 0.717, and 0.718 (all p <0.001) for judging fibrosis stages of S ≥ 2, S ≥ 3, and S = 4 by AFP. APRI levels showed significant positive and moderate correlations with inflammation stages (ρ = 0.445, p < 0.001). AST, GGT, and APRI levels showed significant positive but very weak to weak correlations with fibrosis stages (ρ = 0.137, 0.237, 0.281, p < 0.001).

**Conclusions:**

Serum AFP levels increased as pathological levels of inflammation and fibrosis increased in CHB patients. Our data showed the clinical significance of serum AFP levels in diagnosing liver inflammation and fibrosis. Assessment of liver pathology may be improved by creating a predictive mathematical model by which AFP levels with other biomarkers.

## Background

Hepatitis B virus (HBV) infection is a serious public health problem. The World Health Organization estimates that 2 billion people are exposed to HBV annually and more than 350 million are chronically infected worldwide [1]. While most chronically infected individuals remain asymptomatic carriers, many others develop significant hepatic disease. China reports having 93 million HBV carriers and 20 million people infected with chronic hepatitis B (CHB) [2]. In China, 80% hepatocellular carcinoma (HCC) is HBV-related, and more than 350 000 patients diagnosed annually [1]. CHB is a slow, progressive disease characterized by liver fibrosis [3]. Host immune response and HBV gene mutations may influence the development and severity of liver fibrosis [4].

Liver biopsy has been widely accepted as the gold standard for diagnosing and grading liver inflammation and fibrosis [5]. Liver fibrosis is the natural wound healing process of necroinflammation caused by chronic HBV infection, and the pathogenic process leading to cirrhosis [6]. Detecting liver fibrosis non-invasively in CHB patients is difficult, and even with limitations like sampling variations, observer error and complications [7], biopsy is still recommended for assessing fibrosis severity and identifying patients at risk for HCC and cirrhosis [8]. Biopsy also differentiates immune tolerance from failed immune clearance, which may predict a poorer response to antiviral treatment [6]. However, non-invasive methods originally validated in patients with chronic hepatitis C, are being used increasingly to evaluate CHB. Biological approaches quantifying biomarkers in serum samples [7] or physical approaches applying ultrasound or magnetic resonance to assess liver stiffness [8] have been used to complement liver biopsy. Serum biomarkers of liver inflammation and fibrosis include indirect markers, such as prothrombin index, platelet count and aspartate aminotransferase (AST)/alanine aminotransferase (ALT) ratio, and AFP levels, all associated with fibrosis [9,10]; or direct markers, including hyaluronic acid [11], matrix metalloprotein (MMP) [7], and collagens such as procollagen III and collagen IV [9,10] that directly reflect physical properties of the hepatic extracellular matrix. Advantages of these tests include availability and reproducibility across laboratories [12]; the main disadvantage is that they are not liver-specific and results may be influenced by comorbid conditions, requiring critical review for false positive and false negative results [9,10].

AFP is a fetal protein produced in the yolk sac and liver of the developing fetus. Its molecular weight is between that of albumin and α1-globulin, suggesting an early form of albumin [13]. Serum AFP was reported to be a tumor marker for HCC as early as 1963 [14]. Although AFP is not specific for HCC, elevated AFP levels are seen in chronic liver disease, especially viral hepatitis, and non-hepatic malignancies such as pancreatic, gastric, biliary and germ cell tumors [13]. Determination of AFP levels has been used to monitor HCC onset and progression, evaluate effectiveness of curative treatment, and predict outcomes [15]. AFP is a useful screening tool for HCC in developing countries where HCC prevalence is high and CHB infection is its major risk factor [16]. High levels of AFP (>400 ng/ml) are strongly predictive for HCC. AFP can be used indirectly as an index to indicate fibrosis stage in chronic hepatitis C virus infection [17,18]. However, it is not clear if AFP levels are correlated with ongoing liver damage and repair in chronic liver disease such as CHB.

Few studies have been conducted to evaluate liver cirrhosis by analyzing AFP levels or evaluating associations between AFP levels and fibrosis stages. We hypothesized that analyzing serum AFP levels at different stages of inflammation and fibrosis would clarify the diagnostic value of AFP in predicting the grade and stage of liver pathology. This study aimed to determine the subclinical significance of low serum AFP levels by investigating the relationship between serum AFP levels and different inflammation and fibrosis stages in CHB patients.

## Methods

### Patients

This retrospective cross-sectional study included a total of 619 consecutive patients older than 12 years with CHB who were admitted to the First Affiliated Hospital of Fujian Medical University from March 2005 to December 2011. CHB was defined as HBsAg positivity for at least 6 months, serum HBV DNA above 2000 IU/ml (or 10^4^ copies/ml), and alanine aminotransferase (ALT) levels above the upper limit of normal, 40 IU/ml.

Patients with other types of viral hepatitis, alcoholic liver disease, decompensated cirrhosis, autoimmune hepatitis, concurrent infection with human immunodeficiency virus (HIV), hereditary liver diseases, and drug-induced liver injury were excluded. Based on the 2009 AASLD recommendations, initial screening for HCC should be done using B-mode ultrasound, and MRI scan and enhancement should be adopted to exclude canceration of liver nodules when necessary. In this study, all study subjects underwent at least one B-mode ultrasound examination prior to liver biopsy. Patients with AFP levels > 200 ng/ml underwent CT/MRI scan with enhancement, and all patients diagnosed with hepatocellular carcinoma were excluded. Patients with moderate to severe fatty liver (as revealed by B-mode ultrasound) were also excluded to prevent any possible bias. All included patients had not received any antiviral therapy prior to liver biopsy. Serum and liver biopsy samples were collected from all patients within 7 days after hospital admission.

The internal review board of First Affiliated Hospital of Fujian Medical University reviewed and approved the study protocol. Enrolled patients were deidentified, only their applicable data were reviewed, so signed informed consent was waived.

### Methods

#### Liver biopsy and histopathology

**Figure 1:**
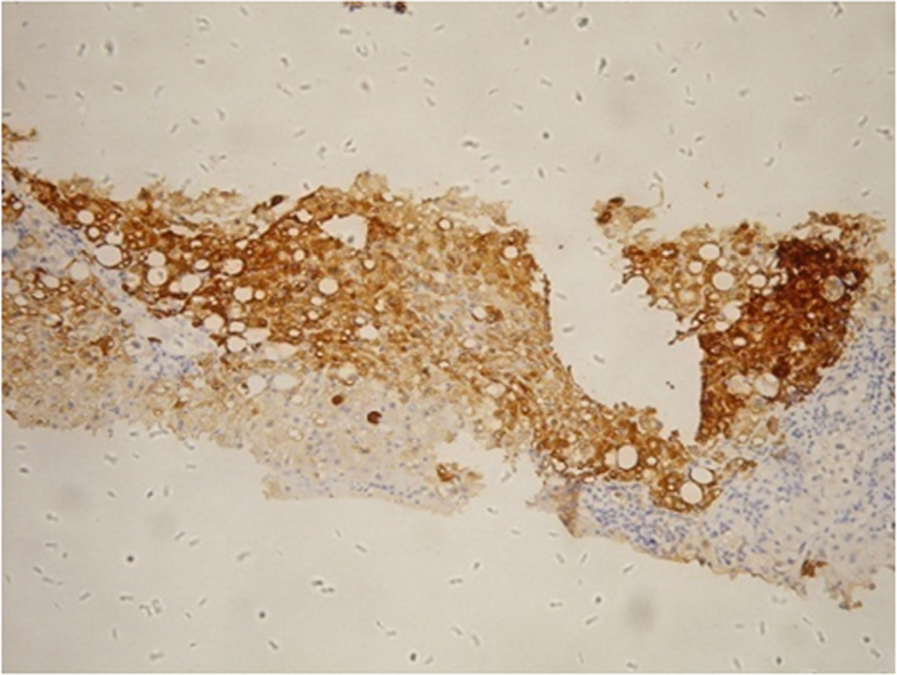
**Positive HBcAg signals on CHB patient biopsy.** Detection of HBcAg levels by anti-HBc as first antibody using immunohistochemical staining, 100X magnification.

All patients underwent liver biopsy using the Cardinal 18G Tru-Cut® Biopsy Needle (CardinaHealth; Dublin, OH) under the guidance of Doppler ultrasound (ACUSON Aspen Color Doppler Ultrasound, Siemens Medical Solutions, Malvern, PA, USA). All biopsies were performed by different experienced doctors, each of whom had performed the procedure more than 20 times. All procedures were performed under the guidance of a B-mode ultrasound. Hepatic tissue samples which were 1.5- 2 cm in length or longer, were fixed in 4% neutral formalin, paraffin imbedded, and serially sectioned to stain with Hematoxylin-Eosin (HE), collagen, reticulum fiber and immunohistochemical staining. Collagen staining was done by Van Gieson staining and the reticulum fiber staining was done by Gomori staining (IHC World Life Science Products, Woodstock, MD, USA). Standard indirect labeling techniques for immunohistochemical staining were applied using antibodies against components of HBsAg and HBcAg as included in the 9000 Polymer Detection System for Immuno-Histological Staining (GBI Company, USA) (Figure 1). Antibodies were detected with 3-amino-9-ethyl-carbazole substrate kit (Zymed Laboratories, Inc. USA). All films were independently reviewed by at least two pathologists.

Pathological diagnosis of liver disease was based on the Programme of Prevention and Cure for Viral Hepatitis, which was developed by the Society of Infectious Diseases and Parasitology and the Chinese Society of Hepatology of the Chinese Medical Association and has been widely used in China since 2000 [19]. Patients’ biopsy samples were classified separately into 5 levels of inflammation (G) and fibrosis (S) stages expressed as G 0 ~ 4 and S 0 ~ 4, as previously described [6]. The key features of liver inflammation and fibrosis stages are shown in Table 1.

**Table 1:** Key features of liver inflammation and fibrosis stages*

**Liver inflammation stages(G)**			**Liver fibrosis stages(S)**	
**Stage**	**Portal area**	**In lobule**	**Stage**	**Fibrosis**
**0**	**None**	**None**	**0**	**None**
1	Lymphopoiesis within portal area	Hepatocyte hydropic or ballooning degeneration, with/without spotty or focal necrosis	1	Mild fibrosis without septa
2	Mild piecemeal necrosis	Spotty or focal necrosis, formation of acidophilic bodies	2	Moderate fibrosis with few septa
3	Moderate piecemeal necrosis	Severe degeneration and necrosis,with formation of necrosis bridge	3	Severe fibrosis with numerous septa, but with no sign of false lobules
4	Severe piecemeal necrosis	Severe necrosis bridge,multiple lobular necrosis	4	Formation of false lobules

*Society of Infectious Diseases and Parasitology and Society of Hepatology, Chinese Medical Association (6).

#### Laboratory examinations

Venous blood was collected from all patients to detect and quantify serum AFP. Serum AFP was measured quantitatively by electrochemiluminescence as previously described [20]. Electrochemiluminescence was done using an autoanalyzer (Modular Analytics E170 Immunology Analyzer, Roche Diagnostics, USA), with the normal reference value of 0 ~ 13.6 ng/ml and an upper limit of detection of 1210 ng/ml. The normal AFP level was defined as 0 ~ 15 ng/mL as previously determined [3].

### Statistical analysis

Age was normally distributed and presented as mean with standard deviation. AFP was non-normally distributed and presented as median with inter-quartile range. Other categorical data were presented as count and percentage. Receiver operating characteristic (ROC) curves were employed to obtain areas under the curve (AUC), sensitivity, and specificity. Spearman’s rank correlation coefficient (ρ) was performed to evaluate correlations between AFP, AST, ALT, and GGT versus liver inflammation and fibrosis stages. Two multivariable ordinal regression models were performed to show the associations between AFP, GGT, and APRI (AST/PLT ratio) and inflammation and fibrosis stages, after controlling for age and gender. All statistical assessments were two sided and evaluated at the 0.05 level of significance. Statistical analyses were performed using SPSS 15.0 statistics software (SPSS Inc, Chicago, IL).

## Results

### Demographic characteristics

A total of 619 patients with CHB were recruited into this study. The mean age of the patients was 38.9 years (range from 13 to 71 years). The study population comprised 506 males (81.7%) and 113 females (18.3%), of whom 62.5% and 53.6% had inflammation and fibrosis in stages 3–4, respectively (Table 2). There were no significant differences in AFP levels between males and females either by the two independent samples t-test or the nonparametric Mann–Whitney test (data not shown). Median AFP, AST, ALT and GGT values were 4.7 ng/mL, 63 U/L, 93 U/L and 54 U/L, respectively. The median APRI was 0.41. (Table 2). Representative result of HBV infection is shown in Figure 1.

**Table 2:** Summary of patients’ characteristics

		**N = 619**
**Age**^**1 **^**(years)**		**38.9 ± 11.5**
Gender	Male	506 (81.7%)
	Female	113 (18.3%)
Inflammation stage	0-1	82 (13.2%)
	2	150 (24.2%)
	3	265 (42.8%)
	4	122 (19.7%)
Fibrosis stage	0-1	151 (24.4%)
	2	136 (22.0%)
	3	129 (20.8%)
	4	203 (32.8%)
AFP (ng/ml)^2^		4.7 (2.5, 13.7)
ALT (U/L)^2^		93.0 (46.0, 241.0)
AST (U/L)^2^		63.0 (37.0, 135.0)
GGT (UL)^2^		54.0 (31.0, 98.0)
APRI (AST/PLT ratio)^2^		0.41 (0.21, 0.83)

Two subjects with inflammation stage 0, and 80 subjects with inflammation stage 1 were combined into one group; 27 subjects with fibrosis stage 0, and 124 subjects with fibrosis stage 1 were combined into one group.

^1^Data presented as mean with standard deviation. ^2^Data presented as median with inter-quartile range. Other data are presented as count and percentage.

### Correlations of AFP levels versus liver inflammation and fibrosis stages

**Figure 2:**
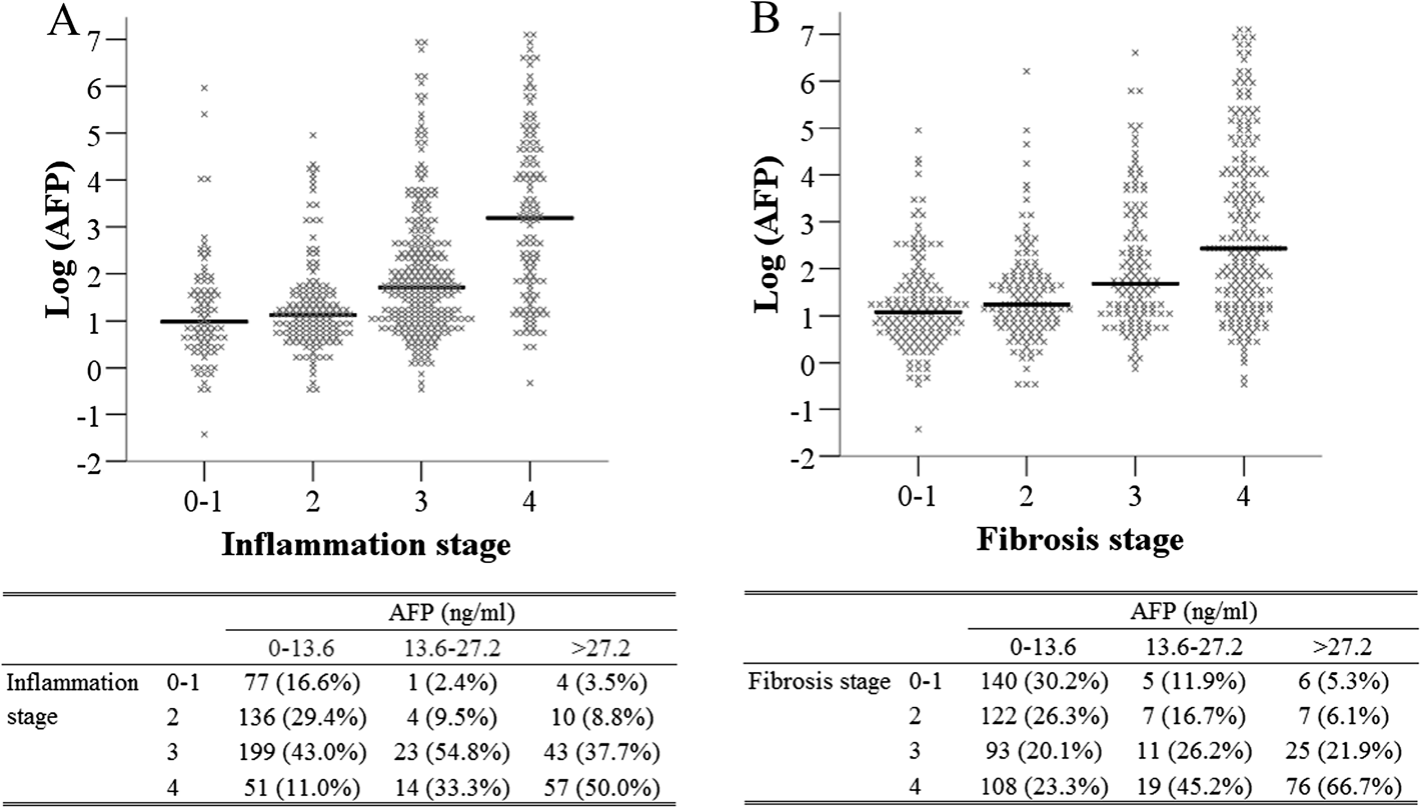
**The distribution of AFP vs. inflammation stage (A) and fibrosis stage (B).** The upper limit of AFP normal range is 13.6 ng/ml, patients were divided into three groups by AFP levels according to normal (0–13.6), 1–2 upper limit of AFP normal range (13.6-27.2), and more than 2 upper limit of AFP normal range (>27.2).

Our data showed that the log AFP median levels increased with inflammation and fibrosis stages (Figure 2). The median values of AFP at liver inflammation stages of G0-1, G2, G3, and G4 were 2.7, 3.1, 5.5, and 24.3 ng/ml, respectively. Spearman’s rank correlation coefficient showed significant positive and moderate correlations between AFP and inflammation stages (ρ = 0.436, p < 0.001). The median values of AFP at liver fibrosis stages S0-1, S2, S3, and S4 were 3.0, 3.4, 5.4, and 11.3 ng/ml, respectively. Spearman’s rank correlation coefficient showed significant positive and moderate correlations between AFP and fibrosis stages (ρ = 0.404, p < 0.001). ALT, AST, and GGT levels also showed significant positive but weak correlations with inflammation stages (ρ = 0.271, 0.393, 0.361, p < 0.001). APRI level showed significant positive and moderate correlations with inflammation stages (ρ = 0.445, p < 0.001). AST, GGT, and APRI levels showed significant positive but very weak to weak correlations with fibrosis stages (ρ = 0.137, 0.237, 0.281, p < 0.001) (Table 3).

**Table 3:** Correlations of AFP, ALT, AST, GGT, and APRI with inflammation and fibrosis stages

		**AFP (ng/ml)**	**ALT (U/L)**	**AST (U/L)**	**GGT (UL)**	**APRI (AST/PLT ratio)**
Inflammation stage	0-1	2.7 (1.6, 5.2)	49.0 (29.0, 94.0)	31.0 (24.0, 58.0)	33.0 (20.0, 62.0)	0.17 (0.11, 0.40)
	2	3.1 (2.1, 4.7)	85.5 (41.0, 155.0)	48.5 (32.0, 85.0)	38.5 (27.0, 63.0)	0.26 (0.15, 0.49)
	3	5.5 (2.9, 13.5)	114.0 (51.0, 284.0)	79.0 (42.0, 148.0)	57.0 (35.0, 96.0)	0.52 (0.27, 0.89)
	4	24.3 (4.9, 108.2)	132.5 (61.0, 306.0)	97.0 (56.0, 172.0)	90.0 (58.0, 144.0)	0.73 (0.42, 1.50)
	Spearman's ρ	0.436*	0.271*	0.393*	0.361*	0.445*
Fibrosis stage	0-1	3.0 (1.9, 5.2)	78.0 (35.0, 171.0)	44.0 (29.0, 88.0)	38.0 (23.0, 64.0)	0.25 (0.13, 0.49)
	2	3.4 (2.3, 6.1)	113.5 (57.5, 281.0)	74.5 (43.5, 136.0)	52.0 (30.0, 83.0)	0.40 (0.21, 0.76)
	3	5.4 (2.9, 16.6)	120.0 (51.0, 324.0)	75.0 (41.0, 169.0)	61.0 (32.0, 106.0)	0.53 (0.22, 1.14)
	4	11.3 (3.8, 59.5)	84.0 (44.0, 187.0)	67.0 (41.0, 131.0)	66.0 (40.0, 118.0)	0.54 (0.29, 1.02)
	Spearman's ρ	0.404*	0.021	0.137*	0.237*	0.281*

Data are presented by median with IQR. *P-value < 0.001.

### Diagnostic value of AFP in dichotomizing liver inflammation stages

After controlling for age and gender, the ordinal regression model showed that higher AFP and APRI levels were significantly associated with higher inflammation stages. The odds of higher stages to lower stages increased with increasing levels of AFP and APRI (odds ratios of 1.60 and 1.88 respectively; both p < 0.001). There was no significant association between GGT levels and inflammation stage (Table 4).

**Table 4:** Multivariable ordinal regression models for inflammation stage and fibrosis stage

	**Ordinal regression model for inflammation stage**			**Ordinal regression model for fibrosis stage**		
	**Coefficient (95% CI)**	**Coefficient (95% CI) in exponential scale**	**P-value**	**Coefficient (95% CI)**	**Coefficient (95% CI) in exponential scale**	**P-value**
Threshold						
stage 0/stage 1-4	−5.16 (−6.96, −3.37)	0.01 (0.00, 0.03)	<0.001	−2.44 (−3.59, −1.29)	0.09 (0.03, 0.27)	<0.001
stage 0–1/stage 2-4	−1.17 (−2.30, −0.03)	0.31 (0.10, 0.97)	0.044	−0.37 (−1.47, 0.73)	0.69 (0.23, 2.07)	0.507
stage 0–2/stage 3-4	0.45 (−0.67, 1.58)	1.57 (0.51, 4.84)	0.432	0.79 (−0.30, 1.89)	2.21 (0.74, 6.61)	0.157
stage 0-3/stage 4	2.96 (1.80, 4.12)	19.32 (6.08, 61.42)	<0.001	1.85 (0.74, 2.95)	6.33 (2.10, 19.12)	0.001
Independent variables						
Age (year)	0.00 (−0.01, 0.02)	1.00 (0.99, 1.02)	0.665	0.02 (0.00, 0.03)	1.02 (1.00, 1.03)	0.011*
Female to male	0.41 (0.01, 0.82)	1.51 (1.01, 2.27)	0.047*	0.23 (−0.17, 0.63)	1.26 (0.85, 1.88)	0.252
logAFP	0.47 (0.35, 0.59)	1.60 (1.42, 1.81)	<0.001*	0.53 (0.40, 0.65)	1.70 (1.50, 1.92)	<0.001*
logGGT	0.14 (−0.10, 0.39)	1.15 (0.90, 1.47)	0.259	−0.11 (−0.35, 0.13)	0.90 (0.71, 1.14)	0.363
logAPRI	0.63 (0.43, 0.83)	1.88 (1.54, 2.29)	<0.001*	0.36 (0.17, 0.55)	1.43 (1.18, 1.73)	<0.001*

*P < 0.05 indicates the corresponding variable was significantly associated to inflammation stage or fibrosis stage. APRI = AST/PLT, AST and ALT were excluded from both models due to its colinearity to APRI.

ROC curve analyses revealed that AUCs were 0.685, 0.727, and 0.755 (all p <0.001) for judging inflammation stages of G ≥ 2, G ≥ 3, and G = 4 by AFP levels, and were 0.741, 0.746, and 0.714 for judging inflammation stages of G ≥ 2, G ≥ 3, and G = 4 by APRI levels. The AUCs of AFP and APRI for judging inflammation stages of G ≥ 2, G ≥ 3, and G = 4 did not differ from each other significantly (all p > 0.05) (Table 5). In addition, Figure 2A showed that 88.1% (54.8 + 33.3) of patients with AFP levels between 13.6-27.2 ng/ml had inflammation stages 3–4, while 50% of those with AFP levels >27.2 ng/ml had inflammation stage 4.

**Table 5:** Summary of the areas under the ROC curves (AUC) for AFP and APRI

		**Inflammation**	**Fibrosis**
		**AUC (95% CI)**	**AUC (95% CI)**
Judging stage ≥ 2	AFP	0.685 (0.647 - 0.722)	0.691 (0.653 to 0.727)
	APRI	0.741 (0.704 - 0.775)	0.687 (0.649 - 0.723)
	P-value	0.074	0.897
Judging stage ≥ 3	AFP	0.727 (0.690 - 0.762)	0.717 (0.680 to 0.752)
	APRI	0.746 (0.709 - 0.779)	0.649 (0.610 - 0.687)
	P-value	0.430	0.007*
Judging stage = 4	AFP	0.755 (0.719 - 0.789)	0.718 (0.681 to 0.753)
	APRI	0.714 (0.677 - 0.750)	0.618 (0.578 - 0.656)
	P-value	0.221	<0.001*

*P < 0.05 indicate a significant difference between AUCs of AFP and APRI.

ROC, receiver operating characteristic; AUC, area under the curve.

### Diagnostic value of AFP in dichotomizing liver fibrosis stages

After controlling for age and gender, the ordinal regression model showed that higher AFP and APRI levels were significantly associated with higher fibrosis stages. The odds of higher stages to lower stages increased with increasing AFP and APRI values (odds ratios of 1.70 and 1.43; p < 0.001). There was no significant association between GGT levels and fibrosis stages (Table 4).

ROC curve analyses revealed that AUCs were 0.691, 0.717, and 0.718 (all p <0.001) for judging fibrosis stages of S ≥ 2, S ≥ 3, and S = 4 by AFP levels. The AUCs of AFP for judging fibrosis stages of S ≥ 3 and S = 4 (0.717 and 0.718, respectively) were significantly higher than those of APRI (0.649 and 0.618, respectively; p = 0.007 and p < 0.001) (Table 5). In addition, 81.4% (26.2 + 45.2) of patients with AFP levels between 13.6-27.2 ng/ml had fibrosis stages 3–4, and 66.7% of those with AFP levels >27.2 ng/ml had fibrosis stage 4 (Figure 2B).

## Discussion

Results of this study indicated that the extent of liver damage in CHB patients had an obvious effect on AFP serum levels. There were significant positive and moderate correlations between AFP and inflammation stages and between AFP and fibrosis stages. AFP also had a significant weak to moderate correlation with AST, ALT and GGT values (Spearman’s correlation coefficients were 0.22, 0.331, and 0.445, respectively; all p < 0.001). Overall, as pathological levels of inflammation and fibrosis increased in patients with CHB, the levels of serum AFP also increased.

Age and gender had little influence on changes in serum AFP levels in this study. Approximately 70% of CHB patients in China are infected via mother-to-child vertical transmission, making it difficult to estimate the median duration of CHB infection. Since the initial detection of CHB infection does not represent the start of infection, we used age to replace the duration of infection. Ninety percent of those infected in infancy, 50% who are infected in childhood, and even fewer over the next years of young adulthood will become chronically infected, with a high life-time risk of developing HCC [21]. Liver pathology helps to determine prognosis, as well as to determine who should receive antiviral therapy [22]. Patients admitted to our medical center were preparing for antiviral therapy and the aim of liver biopsy in these cases was to clarify the pathological status of the active CHB phase. Based on patient selection, inflammation and fibrosis stages increased with age, and so did AFP levels.

We showed that serum APRI values were significantly associated with inflammation stages. We excluded serum AST and ALT values from the multivariate analysis due to its co-linearity with APRI values. Importantly, we found that AFP levels increased as inflammation stages progressed. The median serum AFP values in patients at G0-G3 were within the normal range, while patients at G4 had a significant elevation of AFP median values. The correlation coefficient between AFP levels and inflammation stages (0.436) showed a moderate positive association between AFP levels and inflammation stages in CHB patients. Marked changes in serum AFP are not expected with light liver inflammation (G0 ~ 2). We observed a higher correlation between AFP and inflammation when liver inflammation was more severe.

The specific program of prevention and cure for viral hepatitis that is generally used in China [19] is similar to the METAVIR semiquantitative scoring system [23], and classifies fibrosis into 5 stages: F0 (no fibrosis), F1 (mild fibrosis without septa), F2 (moderate fibrosis with few septa), F3 (severe fibrosis with numerous septa without cirrhosis) and F4 (cirrhosis). CHB patients with significant fibrosis (METAVIR F >2) are prescribed antiviral treatment. In the present study, patients at stages S3 and S4 had a significant elevation in AFP levels, while patients at stages S0, S1 and S2 had normal AFP values. The correlation coefficient between AFP and fibrosis stages was 0.404. AFP levels also showed a moderate positive trend in relationship to fibrosis stages in CHB patients. Investigation of the relationship between AFP levels and liver stiffness using transient elastography showed similar results (correlation coefficient of 0.317 between AFP and fibrosis stages) [8]. These data suggest that AFP plays a role in regeneration of liver tissue, and inflammation and liver fibrosis constitute an indirect index of regeneration. More inflammation is therefore linked with more regeneration, and more liver fibrosis. One critical finding of the present study was that serum AFP levels had a diagnostic value for severity of inflammation and fibrosis even in patients with “normal” levels of serum AFP. Low AFP values in some adult patients still indicated a “severe” condition, reflecting the association between AFP and liver regeneration.

We previously showed that serum ceruloplasmin (CP) levels were negatively and indirectly associated with inflammation and fibrosis, and used serum CP in combination with routinely measured clinical parameters to establish a non-invasive model to predict fibrosis [24]. The prognostic value of a number of other biomarkers for liver fibrosis, such as FibroTest, FibroMeter, FIB-4, ELF, APRI, and FibroScan elastography, were recently investigated [25,26], and only the FibroTest had no significant difference in prognostic value compared to liver biopsy. There has also been a recent focus on developing novel proteomic biomarkers candidates for liver fibrosis in hepatitis C [27]. Although a number of biomarkers including albumin, platelets, hyaluronic acid and AST have been evaluated, there is currently no single marker which successfully predicts significant fibrosis in HBV-related liver disease, and multiple biomarkers are needed to complement clinical data [7]. In the present study, we compared the prognostic value of AFP with that of liver biopsy, using a METAVIR-like scoring system [23]. There was a direct relationship between AFP and both inflammation and fibrosis, suggesting that AFP had a prognostic value, especially given the AUROC scores in our study. The most important criteria for use of a specific non-invasive biomarker in clinical practice is the number of patients correctly classified by the method for a defined end-point based on the reference standard for the method [9]. Based on these criteria, AFP is a promising biomarker to assess liver pathology.

To our knowledge, this is the first study to examine the relationship between serum AFP levels and the pathological status of inflammation and fibrosis in patients with CHB. Nevertheless, this study has some limitations. First, the study was done in a single medical center and the sample was relatively small. Second, we applied the accepted pathological staging system used in China but there are no known comparisons between this system and other universal systems, which may limit the value of our findings. It is important to note that the occurrence of acute flares has an important role in the progression of CHB. Differences in inflammation grade make it difficult to evaluate the performance of fibrosis biomarkers such as AFP which are strongly influenced by inflammation. Finally, serum AFP values in this study were measured at certain transverse sections of time and not dynamically. We are aware that better diagnostic values for liver pathological stages could be attained by repeated and dynamic measurement of AFP levels, and plan to address this issue in our future studies. Values for serum ALT and sonography were not included for each patient, which precludes determining the sensitivity, specificity and overall effectiveness of serum AFP in comparison to other approaches. Future studies with multiple centers and a larger sample size are needed to evaluate the prognostic value of adding AFP to the clinical scoring models currently used [7]. Serum biomarkers have been shown to contribute only modestly to clinical predictive factors for risk assessment, indicating that potential biomarkers must be studied in cohorts with a broad distribution of fibrosis severity [7].

## Conclusion

In conclusion, we showed the subclinical significance of serum AFP levels by analyzing the association between serum AFP and different stages of inflammation and fibrosis. We showed that increasing levels of inflammation and fibrosis in CHB patients were associated with increased serum AFP levels. However, there is no single laboratory parameter that can currently independently predict the prognosis of liver pathology accurately, and an important future goal will be to develop a predictive mathematical model using a combination of different biomarkers.

## Competing interests

The authors declare that they have no competing interests.

## Authors’ contributions

YRL: study concepts, study design. BBL: guarantor of integrity of the entire study, definition of intellectual content. DWZ: literature research, statistical analysis. YYZ: guarantor of integrity of the entire study, data acquisition. JC: statistical analysis. QZ: manuscript editing. JD: study concepts, study design, clinical studies. JJJ: guarantor of integrity of the entire study, manuscript review. All authors read and approved the final manuscript.

## Pre-publication history

The pre-publication history for this paper can be accessed here:

http://www.biomedcentral.com/1471-230X/14/145/prepub

## References

[B1] European Association for the Study of the Liver EASL Clinical Practice Guidelines: management of chronic hepatitis B J Hepatol 2009 50 27 242 10.1016/j.jhep.2008.10.001 19054588

[B2] Liang X Bi S Yang W Wang L Cui G Cui F Zhang Y Liu J Gong X Chen Y Wang F Zheng H Wang F Guo J Jia Z Ma J Wang H Luo H Li L Jin S Hadler SC Wang Y Epidemiological serosurvey of hepatitis B in China—declining HBV prevalence due to hepatitic B vaccination Vaccine 2009 27 6550 6557 19729084 10.1016/j.vaccine.2009.08.048

[B3] Wang D Wang Q Shan F Liu B Lu C Identification of the risk of liver fibrosis on CHB patients using an artificial neural network based on routine and serum markers BMC Infect Dis 2010 10 251 10.1186/1471-2334-10-251 20735842 PMC2939639

[B4] Ding XC Ma LN Li YF Liu XY Zhang X Liu JY Sheng YJ Zhang DZ Hu HD Ren H Association between serum platelet-derived growth factor BB and degree of liver damage, fibrosis and hepatitis B e antigen (HBeAg) Status in CHB patients Hepato Gastroenterol 2012 59 2357 2360 10.5754/hge12388 22688015

[B5] Bravo AA Sheth SG Chopra S Liver biopsy N Engl J Med 2001 344 95 500 10.1056/NEJM200101113440203 11172192

[B6] Chan HL Wong GL Wong VW A review of the natural history of chronic hepatitis B in the era of transient elastography Antivir Ther 2009 14 489 499 19578234

[B7] Park SH Kim CH Kim DJ Suk KT Cheong JY Cho SW Hwang SG Lee YJ Cho M Yang JM Kim YB Usefulness of multiple biomarkers for the prediction of significant fibrosis in chronic hepatitis British J Clin Gastroenterol 2011 45 361 365 10.1097/MCG.0b013e31820d3458 21301354

[B8] Fung J Lai CL Fong DY Yuen JC Wong DK Yuen MF Correlation of liver biochemistry with liver stiffness in chronic hepatitis B and development of a predictive model for liver fibrosis Liver Int 2008 28 1408 1416 10.1111/j.1478-3231.2008.01784.x 18482268

[B9] Castera L Noninvasive methods to assess liver disease in patients with hepatitis B and C Gastroenterol 2012 142 1293 1302 10.1053/j.gastro.2012.02.017 22537436

[B10] Castera L Pinzani M Noninvasive assessment of liver fibrosis: are we ready? Lancet 2010 375 1419 1420 10.1016/S0140-6736(09)62195-4 20417845

[B11] Parsian H Rahimipour A Nouri M Somi MH Qujeq D Assessment of liver fibrosis development in chronic hepatitis B patients by serum hyaluronic acid and laminin levels Acta Ckub Criat 2010 49 257 265 21462814

[B12] Cales P Veillon P Konaté A Mathieu E Ternisien C Chevailler A Godon A Gallois Y Joubaud F Hubert-Fouchard I Oberti F Réaud S Hunault G Mauriat F Lunel-Fabiani F Reproducibility of blood tests of liver fibrosis in clinical practice Clin Biochem 2008 41 10 18 10.1016/j.clinbiochem.2007.08.009 17988658

[B13] Taketa K Alfafetoprotein: reevaluation in hepatology Hepatology 1990 12 1420 1432 10.1002/hep.1840120625 1701754

[B14] Abelev GI Alpha-fetoprotein in ontogenesis and its association with malignant tumors Advan Cancer Res 1971 14 295 358 4107670 10.1016/s0065-230x(08)60523-0

[B15] Lai Q Melandro F Pinheiro RS Donfrancesco A Fadel BA Levi Sandri GB Rossi M Berloco PB Frattaroli FM Alpha-fetoprotein and novel tumor biomarkers as predictors of hepatocellular carcinoma recurrence after surgery: a brilliant star raises again Int J Hepatol 2012 2012 893103 doi:10.1155/2012/893103 22792474 10.1155/2012/893103 PMC3391901

[B16] Yu MW Hsu FC Sheen IS Chu CM Lin DY Chen CJ Liaw YF Prospective study of hepatocellular carcinoma and liver cirrhosis in asymptomatic chronic hepatitis B virus carriers Am J Epidemiol 1997 145 1039 1047 10.1093/oxfordjournals.aje.a009060 9169913

[B17] Bruce MG Bruden D McMahon BJ Christensen C Homan C Sullivan D Deubner H Williams J Livingston SE Gretch D Clinical significance of elevated alpha-fetoprotein in Alaska Native patients with chronic hepatitis C J Viral Hepatol 2008 15 179 187 10.1111/j.1365-2893.2007.00928.x 18233991

[B18] Livingston SE Deubner H Bruden DL McMahon BJ Homan CE Townshend-Bulson LJ Bruce MG Hennessy TW Williams JL Gretch DR Factors associated with the progression of fibrosis on liver biopsy in Alaska Native and American Indian persons with chronic hepatitis C Can J Gastroenterol 2010 24 445 451 20652161 10.1155/2010/692036 PMC2918486

[B19] Society of Infectious Diseases and Parasitology and Chinese Society of Hepatology of Chinese Medical Association The programme of prevention and cure for viral hepatitis Zhonghua Ganzangbing Zazhi 2000 8 324 329

[B20] Demirturk F Caliskan AC Aytan H Sahin S A preliminary retrospective study about the relationship between ductus venosus Doppler indices, nuchal translucency (NT) and biochemical markers in the first and second trimester screening tests Gynecol Endocrinol 2012 28 378 381 10.3109/09513590.2011.631633 22364171

[B21] Kew MC Hepatocellular carcinoma in developing countries. Prevention, diagnosis and treatment World J Hepatol 2012 4 99 104 10.4254/wjh.v4.i3.99 22489262 PMC3321496

[B22] Lok AS McMahon BJ Chronic hepatitis B: update 2009 Hepatology 2009 50 661 662 10.1002/hep.23190 19714720

[B23] Bedossa P Poynard T An algorithm for the grading of activity in chronic hepatitis C. The METAVIR Cooperative Study Group Hepatology 1996 24 289 293 10.1002/hep.510240201 8690394

[B24] Zeng DW Liu YR Zhang JM Zhu YY Lin S Yu J Li YB Chen J Zheng Q Jiang JJ Dong J Serum ceruloplasmin levels correlate negatively with liver fibrosis in males with chronic Hepatitis B: a new noninvasive model for predicting liver fibrosis in HBV-related liver disease PLoS One 2013 8 e77942 10.1371/journal.pone.0077942 24282481 PMC3837017

[B25] Poynard T Ngo Y Perazzo H Munteanu M Lebray P Moussalli J Thabut D Benhamou Y Ratziu V Prognostic value of liver fibrosis biomarkers: A meta-analysis Gastroenterol Hepatol 2011 7 445 454 PMC3264893 22298979

[B26] Baranova A Lai P Birerdinc A Younossi ZM Non-invasive markers for hepatic fibrosis BMC Gastroenterol 2011 11 91 10.1186/1471-230X-11-91 21849046 PMC3176189

[B27] Gangadharan B Bapat M Rossa J Antrobus R Chittenden D Kampa B Barnes E Klenerman P Dwek RA Zitzmann N Discovery of novel biomarker candidates for liver fibrosis in hepatitis C patients: A preliminary study PLoS One 2012 7 e39603 10.1371/journal.pone.0039603 22761838 PMC3383672

